# Case report: A compound heterozygous mutations in *ASNS* broadens the spectrum of asparagine synthetase deficiency in the prenatal diagnosis

**DOI:** 10.3389/fped.2023.1273789

**Published:** 2023-10-13

**Authors:** Linyan Zhu, Yixi Sun, Yuqing Xu, Pengzhen Jin, Huiqing Ding, Minyue Dong

**Affiliations:** ^1^ Department of Obstetrics and Gynaecology, The First Affiliated Hospital of Ningbo University, Ningbo, China; ^2^ Women’s Hospital, School of Medicine, Zhejiang University, Hangzhou, China; ^3^ Key Laboratory of Reproductive Genetics, Ministry of Education (Zhejiang University), Hangzhou, China

**Keywords:** ASNS, prenatal diagnosis, asparagine synthetase deficiency, whole-exome sequencing, case report

## Abstract

Asparagine synthetase deficiency (ASNSD) is a rare congenital disorder characterized by severe progressive microcephaly, global developmental delay, spastic quadriplegia, and refractory seizures. ASNSD is caused by variations of the ASNS gene. The present study showed a Chinese family with a fetus suffering microcephaly. Whole-exome sequencing and Sanger sequencing were used to identify the disease-associated genetic variants. Compound heterozygous variants c.97C>T p. (R33C) and c.1031-2_1033del were identified in the ASNS gene and the variants were inherited from the parents. The mutation site c.97C>T was highly conserved across a wide range of species and predicted to alter the local electrostatic potential. The variant c.1031-2_1033del was classified pathogenic. However, there is no case report of prenatal diagnosis of ASNSD. This is the first description of fetal compound mutations in the ASNS gene leading to ASNSD, which expanded the spectrum of ASNSD.

## 1. Introduction

Asparagine synthetase deficiency (ASNSD, OMIM 615574), a rare autosomal recessive neurometabolic disorder, is characterized by a triad of congenital progressive microcephaly, profound developmental delay, and axial hypotonia followed by spastic quadriplegia (1–4). ASNS gene plays a unique role in brain development and its mutations may result in fetal death or stillbirth (1). The affected individuals typically do not acquire any developmental milestones (2). To date, mutations in the ASNS gene have been reported in children or newborns from a variety of ethnic origins but not in fetuses (3, 5–16).

ASNSD has been reported in newborns or children with either homozygous or compound heterozygous mutations in the *ASNS* gene on chromosome 7q21 (2). The gene encodes asparagine synthetase enzyme which is involved in the synthesis of asparagine (7, 17). The ASNS protein (561 aa) is expressed in the brain, pancreas, thyroid and testes and liver. The adult brain expresses particularly high levels asparagine synthetase (18). However, only some patients had lower Asn levels in plasma and cerebral spinal fluid, which prevented diagnosis on biochemical bases (11).

In the current investigation, we report a case of fetus with severe brain dysplasia of a non-consanguineous couple. Prenatal whole-exome sequencing followed by Sanger sequencing identified compound heterozygous mutations in the ASNS gene, and therefore ASNSD was prenatally diagnosed. This is the first description of fetal compound mutations in the ASNS gene leading to ASNSD, which expanded the spectrum of ASNSD.

## 2. Case presentation

**Figure 1:**
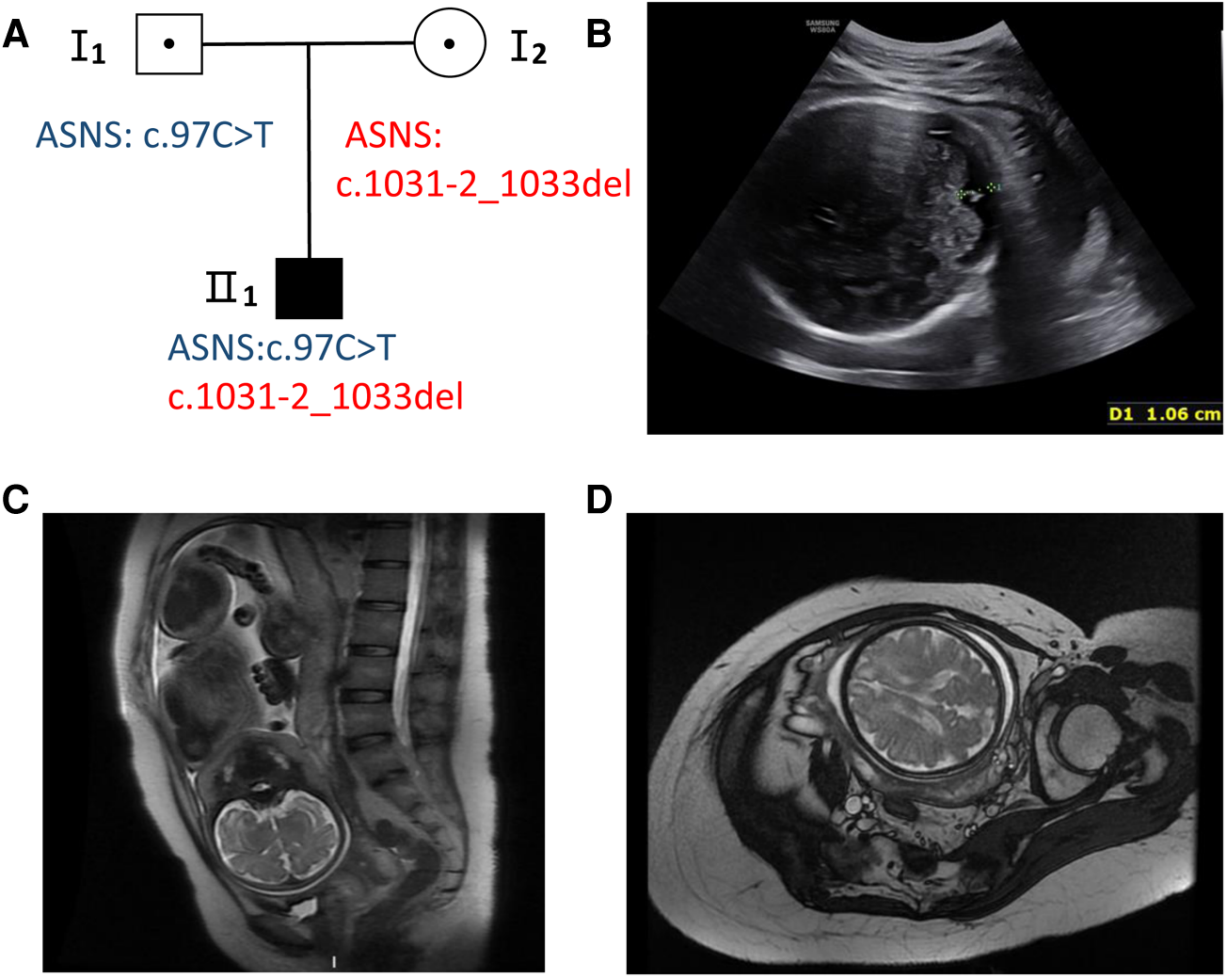
Pedigree and fetal ultrasound scan and MRI findings. (**A**) Pedigree of the family. The solid square (male) represent the affected fetus. (**B**) Fetal ultrasound scan showed the fetal biparietal diameter was 8.3 cm at 37 weeks gestation (II 1). (**C,D**) The fetal brain MRI showed white matter abnormalities with frontal prominence, thinner white matter.

A 31-year-old healthy woman was referred to our clinic due to fetal encephalodysplasia (Figure 1A). Ultrasound scans of the fetus showed reduced biparietal diameter (5.1 cm and 8.3 cm respectively at the 24th and 37th week of gestation) and intrauterine growth retardation (Figure 1B). Fetal magnetic resonance imaging (MRI) at 37th weeks revealed thinner white matter in the temporal and frontal parietal regions on both sides of the fetus, and patchy white matter areas of the subfrontal cortex on both sides (Figures 1C,D). The biparietal diameter, head circumference, and femur length of the fetus were smaller than the gestational age-equivalents (86.1 mm, 299.2 mm, and 67.1 mm, respectively).

## 3. Methodology

To explore the possible genetic cause, we performed CMV to analyze the fetal umbilical blood, but the results did not reveal any disease-causing CNVs >100 kb, implying that the fetal encephalodysplasia might be caused by a single pathogenic gene mutation. To test this hypothesis, WES was carried out to analyze the DNA samples of the fetus and the parents. In the pedigree, we identified variants of c.97C>T p. (R33C) and c.1031-2_1033del in the ASNS (NM_133436.3) gene.

**Figure 2:**
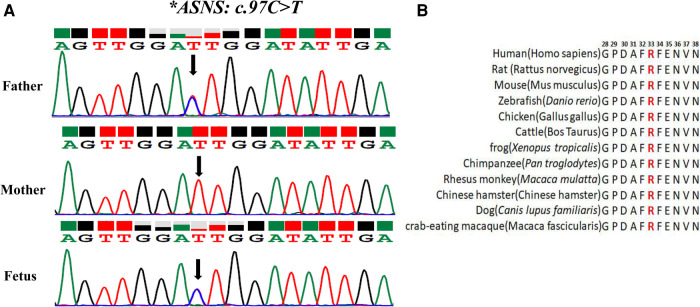
Analysis of the c.97C>T variant in the ASNS gene (**A**) sanger sequencing of the compound heterozygous variants. The black arrow refers to the variant of c.97C>T. (**B**) Conservation status among orthologs.

The variant of c.97C>T was paternally inherited and led to amino acid sequence modifications of p. (R33C) (amino acid arginine to cysteine) (Figure 2A), which was not found in HGMD with a minor allele frequency (MAF) of 0.000003977. ClinVar database rated it as possibly pathogenic. The results of Mutation Taster, PROVEAN, SIFT, and PolyphEN-2 software were all predicted to be harmful or possibly harmful. The mutation was rated as “variants of uncertain significance” according to the ACMG guidelines (2015).

To test the evolutionary conservation of the p. (R33C) amino acid in WT *ASNS*, we compared wild-type amino acid sequence of *ASNS* across 12 different species The results demonstrated that the amino acid was reported to be highly conserved across all 12 species, suggesting that the amino acid plays crucial role in ASNS in the course of evolution and the maintenance of biochemically relevant activities (Figure 2B).

**Figure 3:**
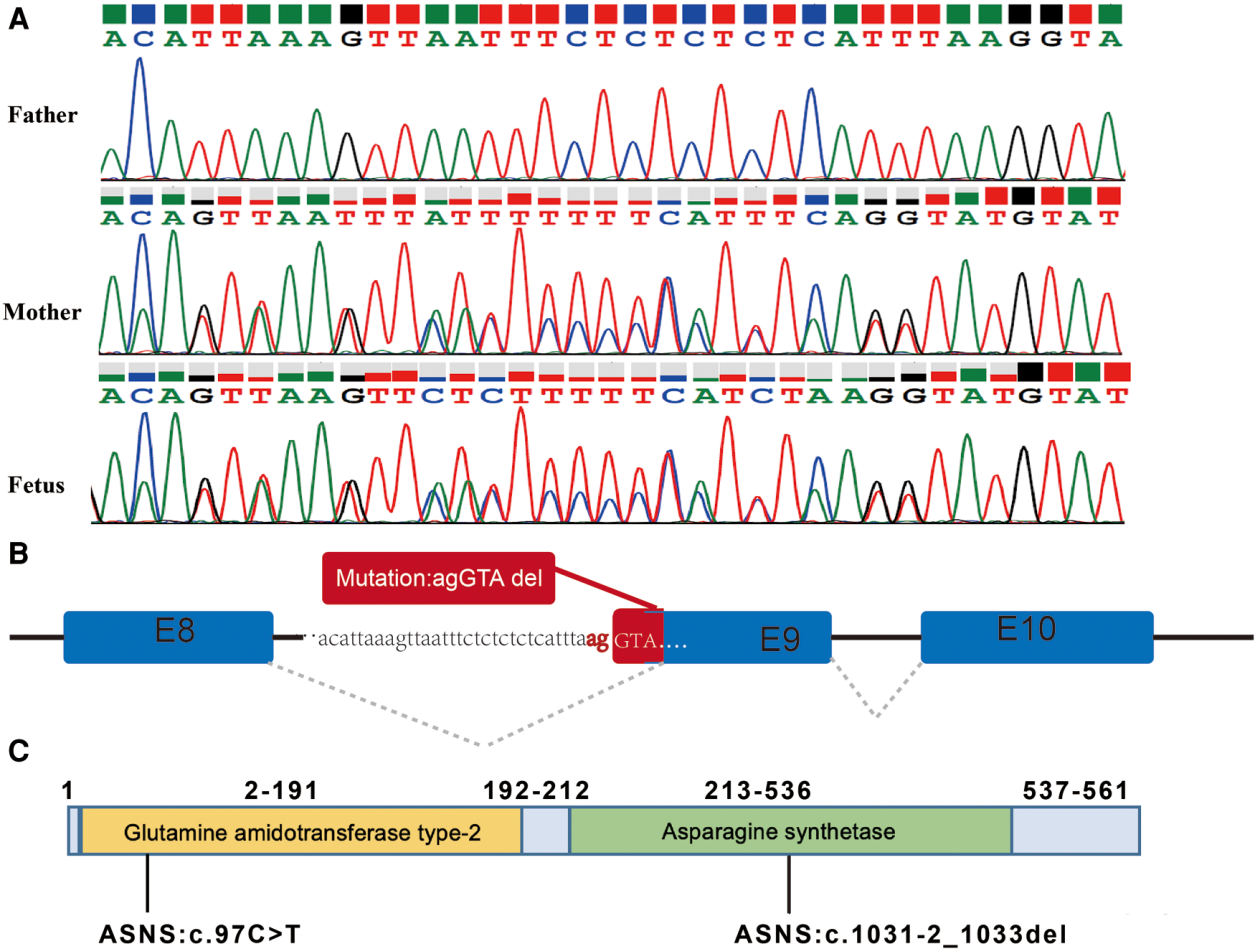
The splicing variant in the ASNS gene and the locations of the variants. (**A**) Variant c.1031-2_1033del identified by Sanger sequencing. (**B**) Splicing model schematic representation. (**C**) The variants of c.97C>T and c.1031-2_1033del are located in the Glutamine amidotransferase type-2 and Asparagine synthetase domains of the ASNS protein, respectively.

The variant of c.1031-2_1033del was maternally inherited and had a deletion of 5 nucleotides at the canonical splicing sites (Figures 3A,B), which was predicted to impair protein function. According to the ACMG and the AMP guidelines, the mutation of c.1031-2_1033del was predicted to be likely pathogenic. The variants of c.97C>T and c.1031-2_1033del are located in the Glutamine amidotransferase type-2 and Asparagine synthetase domains of the ASNS protein, respectively (Figure 3C).

**Figure 4:**
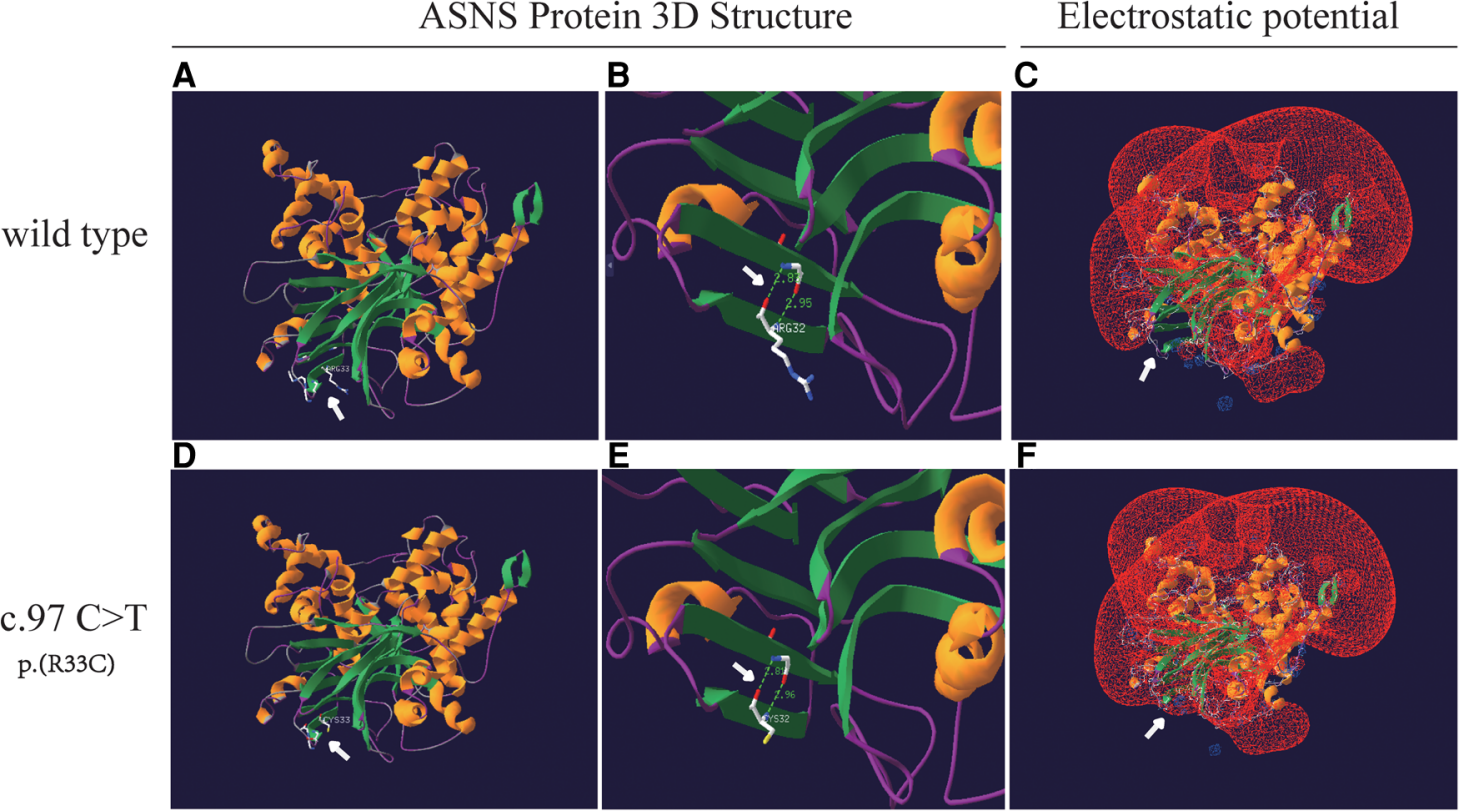
Structure of the ASNS protein. (**A** and **D**) The structure of wild-type and c.97C>T splicing mutation ASNS proteins as predicted by the Swiss-PdbViewer 4.1.0 respectively. (**B** and **E**) Hydrogen bonds are shown as dotted lines and the distances are indicated in the Glutamine amidotransferase type-2 domain (residues 2–191) of the wild-type and splicing mutation ASNS proteins. (**C** and **F**) The local electrostatic potential in wild-type of the ASNS protein (**C**). The ASNS c.97C>T splicing mutation altered the distances between Hydrogen Bonds and the electrostatic potential (**F**).

An analysis of missense variant and the prediction of its deleterious effect were performed by homology modeling in DeepView/Swiss-PdbViewer using the most similar structures available in the Protein Data Bank. Protein structure 3D modeling was performed using Swiss-PdbViewer 4.1.0 (Swiss Institute of Bioinformatics, http://spdbv.vital-it.ch/) (19). The primary sequence of each candidate protein was loaded in Swiss-PdbViewer and aligned onto suitable modeling templates retrieved from SWISS-MODEL. The wild-type and c.97C>T splicing mutation ASNS proteins were superposed in three-dimensional (3D) space using Swiss-PdbViewer 4.1.0 (Figures 4A,D). The Glutamine amidotransferase type-2 domain (residues 2–191) of the wild-type and splicing mutation ASNS proteins is shown in Figures 4B,E. The ASNS c.97C>T splicing mutation altered the protein structure, especially the distances between Hydrogen Bonds and the electrostatic potential (Figures 4C,F).

The pregnancy was terminated at 37th week after the fetal genetic diagnosis, MRI and thorough genetic counseling.

The use of medical information was approved by the Ethics Committee of Women's Hospital, School of Medicine Zhejiang University (IRB-20210230-R) and conformed to the Declaration of Helsinki. All participants provided their written informed consents.

## 4. Discussion and conclusions

So far, over 50 mutations in the ASNS gene have been reported to be associated with ASNSD (20–23) and 101 ASNSD cases have been reported in Chinese and English literatures, most of which are due to recessive missense mutations (4, 21). Among affected children, 71.9% (41/57) died before the age of one. Most surviving children exhibit symptoms such as intractable epilepsy and severe developmental delay (10).

ASNS catalyzes aspartate to asparagine via ATP, producing glutamate and ammonia from its N-terminal. ASNS loss results in reduced cellular asparagine levels critical for early neurological development (2, 24). Common clinical manifestations of ASNSD include microcephaly, severe psychomotor developmental delay, cortical blindness, hyperreflexia, encephalatrophy, increased limb muscle tone, decreased trunk muscle tone, feeding difficulties, and seizures (9, 18, 25). Almost all the cases in newborns or children had microcephaly. In the current investigation, fetal ultrasound and MRI showed that the fetal biparietal diameter was stunted and small for 2 weeks. As these phenotypes are difficult to detect in prenatal fetus, the symptoms of intellectual disability, epilepsy and progressive cerebellar atrophy cannot be observed prenatally. Therefore, it is difficult to diagnose ASNSD in the third trimester of pregnancy. Herein, we diagnosed an ASNSD fetus with the assistance of WES, MRI and ultrasound at the 37th week of gestation.

Hitherto, no case of prenatal diagnosis of ASNSD has been reported previously. There may be several reasons for this. Firstly, the diagnosis of ASNSD mainly depends on the clinical manifestations and the US or MRI after birth, half of the patients reported so far in the literature died during their first years and the others had poor neurological outcomes (11). Due to the limited clinical manifestations of the fetus *in utero*, the symptoms of intellectual disability, epilepsy and progressive cerebellar atrophy cannot be detected prenatally. Secondly, there is currently no specific biochemical examination method for the diagnosis of ASNSD, early detection and prenatal diagnosis of ASNSD may be valid tools to prevent the birth of affected. Previous reports have suggested that ASNSD should be considered in children with congenital microcephaly. This study suggests that fetuses with abnormal brain development and progressive small biparietal diameter should also be considered for this disease and undergo corresponding genetic examinations.

It has been reported that 81.94% of the ASNSD patients had ASNS gene missense variants (21). The variant c.97C>T (p.R33C) site occurres at the high conservation regions (Figure 2B). Protein function prediction analysis showed that c.97C>T caused the 33rd amino acid to change from arginine to cysteine, affecting the protein structure by changing the distance between hydrogen bonds and electrostatic potential. Previous studies have shown that field effect (electrostatic potential) rather than steric effect (volume) played a crucial role in determining abundance of amino acids (26). So, the ASNS c.97C>T mutation may affect protein function by changing the field effect, yet the exact mechanisms require further study.

In conclusion, we report the first case of a fetus diagnosed with ASNSD, which enriched the clinical phenotype of the disease. For fetuses with progressive small biparietal diameter or abnormal brain development, the disease should be considered, and genetic testing should be performed promptly.

## Data Availability

The data that support the findings of this study are available from the corresponding author HD and MD, upon reasonable request.
